# Chicken Anti*-Campylobacter* Vaccine – Comparison of Various Carriers and Routes of Immunization

**DOI:** 10.3389/fmicb.2016.00740

**Published:** 2016-05-19

**Authors:** Patrycja A. Kobierecka, Agnieszka K. Wyszyńska, Jerzy Gubernator, Maciej Kuczkowski, Oskar Wiśniewski, Marta Maruszewska, Anna Wojtania, Katarzyna E. Derlatka, Iwona Adamska, Renata Godlewska, Elżbieta K. Jagusztyn-Krynicka

**Affiliations:** ^1^Department of Bacterial Genetics, Institute of Microbiology, Faculty of Biology, University of WarsawWarsaw, Poland; ^2^Department of Lipids and Liposomes, Faculty of Biotechnology, University of WrocławWrocław, Poland; ^3^Department of Epizootiology and Clinic of Birds and Exotic Animals, Faculty of Veterinary Medicine, Wrocław University of Environmental and Life SciencesWrocław, Poland; ^4^Department of Animal Physiology, Institute of Zoology, Faculty of Biology, University of WarsawWarsaw, Poland

**Keywords:** liposomes, GEM particles, *in ovo* immunization, rCjaAD, *Campylobacter*

## Abstract

*Campylobacter* spp, especially the species *Campylobacter jejuni*, are important human enteropathogens responsible for millions of cases of gastro-intestinal disease worldwide every year. *C. jejuni* is a zoonotic pathogen, and poultry meat that has been contaminated by microorganisms is recognized as a key source of human infections. Although numerous strategies have been developed and experimentally checked to generate chicken vaccines, the results have so far had limited success. In this study, we explored the potential use of non-live carriers of *Campylobacter* antigen to combat *Campylobacter* in poultry. First, we assessed the effectiveness of immunization with orally or subcutaneously delivered Gram-positive Enhancer Matrix (GEM) particles carrying two *Campylobacter* antigens: CjaA and CjaD. These two immunization routes using GEMs as the vector did not protect against *Campylobacter* colonization. Thus, we next assessed the efficacy of *in ovo* immunization using various delivery systems: GEM particles and liposomes. The hybrid protein rCjaAD, which is CjaA presenting CjaD epitopes on its surface, was employed as a model antigen. We found that rCjaAD administered *in ovo* at embryonic development day 18 by both delivery systems resulted in significant levels of protection after challenge with a heterologous *C. jejuni* strain. In practice, *in ovo* chicken vaccination is used by the poultry industry to protect birds against several viral diseases. Our work showed that this means of delivery is also efficacious with respect to commensal bacteria such as *Campylobacter*. In this study, we evaluated the protection after one dose of vaccine given *in ovo*. We speculate that the level of protection may be increased by a post-hatch booster of orally delivered antigens.

## Introduction

Vaccination is commonly recognized as the most effective strategy to prevent human infectious diseases caused by bacterial and viral pathogens. Several human intestinal diseases, including campylobacteriosis, are zoonoses — human diseases acquired by contact with, or by consumption of, contaminated animal products. *Campylobacter jejuni*/*coli* infections are the leading bacterial cause of diarrhoeal illnesses in humans in both developing and developed countries (Kaakoush et al., 2015). Many epidemiological studies indicate that improperly prepared meat from chickens that carry a high load of *Campylobacter* in their intestinal tracts is the key source of human infections (Hermans et al., 2012; Agunos et al., 2014; Zambrano et al., 2014). The mortality connected with *Campylobacter* infections is low and campylobacteriosis is largely a self-limiting disease. However, specific treatment is required for patients infected with strains resistant to clinically important antibiotics such as fluoroquinolones and macrolides (Gibreel and Taylor, 2006; Alfredson and Korolik, 2007; Oh and Jeon, 2015) and for patients who develop neurological symptoms or bacteremia due to infection. As the average life span of Europeans steadily increases, one can expect a greater incidence of serious complications from *Campylobacter* infections, especially in cases affecting older patients. Also, the high social and economic costs of disease cannot be ignored.

While much effort has been made to improve the biosecurity of poultry flocks and hygiene during the processing of poultry products (Katsma et al., 2007; Newell et al., 2011; Wagenaar et al., 2013; Robyn et al., 2015), EFSA data show that since 2005 the level of chicken contamination at farms and the number of reported human campylobacteriosis cases have continued to be high and generally unchanged in many European countries. The number of reported confirmed cases of human campylobacteriosis in the EU in 2013 was 214,779 (EFSA and ECDC, 2015). In 2013, overall, more than 30% of fresh broiler meat samples tested were *Campylobacter* positive, although there were large differences among different countries (EFSA and ECDC, 2014). It is estimated that about 30% of human campylobacteriosis cases in the European Union are associated with consumption or preparation of poultry meat (Wagenaar et al., 2013). Thus, interventions to reduce the level of bird contamination by *Campylobacter* are greatly needed to help solve an important public health concern and limit the incidence of campylobacteriosis in humans. An efficient chicken vaccine against *Campylobacter*, suitable for routine use at poultry farms, would solve this public health problem, but this goal has remained out of reach. Chickens are slaughtered at an age of about 6 weeks. Thus, they have to be vaccinated soon after hatching, when their immune system is still immature, and the presented maternal antibodies may block the immune system induction by an administered vaccine prototype (Sahin et al., 2001; Larson et al., 2008; Shoaf-Sweeney et al., 2008).

The choice of an antigen for subunit vaccine generation is a critical step. Significant criteria to consider when searching for potent antigens are the immunogenicity of an antigen, its localization on live bacteria, and its importance in the pathogen-host interaction and virulence. Rapid advances in the global genetic analysis of *Campylobacter* cells, as well as a deepening understanding of pathogen interactions with eukaryotic cells, has resulted in identification of new antigens that are potentially useful for vaccination (Nielsen et al., 2012; Backert et al., 2013; Hoppe et al., 2014). Numerous *Campylobacter* extracytoplasmic, conserved proteins have been tested as candidates for vaccine development, with delivery via oral, nasal and parenteral routes. Of these, the most thoroughly examined proteins have been CjaA (solute binding protein; component of the ABC transport system), CjaD (peptidoglycan-binding protein), FlaA (flagellin) and CmeC (the outer-membrane component of CmeABC multidrug eﬄux pump). However, the amount of protection obtained varied greatly. The median reduction in *C. jejuni* cecal contents ranged from 6 log10 to less than 1 log10 (Wyszyńska et al., 2004; Buckley et al., 2010; Zeng et al., 2010; Layton et al., 2011; Clark et al., 2012; Neal-McKinney et al., 2014). While these results are not directly comparable, due to many important experimental design differences, it is generally agreed that surface-located, immunodominant proteins are more potent antigens because they are easily accessible for induced antibodies. Thus, the majority of antigens tested have been extracellular proteins. However, immunization with SodB (superoxide dismutase), which is a cytoplasmic protein, also resulted in a modest reduction of colonization (Chintoan-Uta et al., 2015). In order to overcome problems arising from the genetic diversity of *Campylobacter* strains and to strengthen immunization efficacy, hybrid proteins such as CjaA presenting CjaD epitopes on its surface, or recombinant proteins composed of selected parts of surface–exposed colonization proteins (CadF-FlaA-FlpA) (named SECPs), have also been assessed (Neal-McKinney et al., 2014; Kobierecka et al., 2016). The efficacy of subunit vaccines is determined not only by the antigens in the vaccine but also by the delivery vectors, route and scheme of immunization that are used. So, a wide array of delivery strategies has been examined. Usually recombinant proteins have been delivered via intramuscular or subcutantaneous injection, alone or with an adjuvant. The observed protection, mainly against homologous *C. jejuni* strain colonization, varied from no reduction to a modest to significant reduction of colonization, and often the protection effect has varied substantially between individual birds (Zeng et al., 2010; Theoret et al., 2012; Neal-McKinney et al., 2014). It should also be noted that although a majority of experiments resulted in a significant antibody response, generally there has been no correlation between protection and the induced level of specific IgY (Neal-McKinney et al., 2014; Chintoan-Uta et al., 2015).

As *Campylobacter* colonizes chicken caeca, it seems that antibody-independent effectors such as mucins or defensins play a role in protection against colonization (van Dijk et al., 2007; Naughton et al., 2013, 2014). However, it cannot be rule out that intestinal sIgA are also important. Thus, several immunogenic proteins have been delivered orally using attenuated *Salmonella* strains as delivery vehicles (Wyszyńska et al., 2004; Buckley et al., 2010; Layton et al., 2011; Theoret et al., 2012). It should also be noted that oral of vaccination is more feasible to put into practice than injections. However, these experiments produced widely varying levels of protection, even when the same antigen was used. The observed discrepancies may come from the varying schemes of immunization, and more likely from the various chicken lines used for experiments and the differences in their gut microbiota (Rubin et al., 2010; Pan and Yu, 2014; Indikova et al., 2015). In addition to attenuated *Salmonella* strains, an *Eimeria* strain that produces *Campylobacter* CjaA protein has been investigated as a delivery vehicle for chicken immunization against two pathogens (Clark et al., 2012). Comparison of the results of immunization using the same antigens delivered in different ways shows the importance of the route of vaccination. Parenteral immunization with hybrid GST-CjaA or Dps (DNA-binding protein from starved cells) resulted in no protection whereas the same antigens administered orally using *Salmonella* vectors reduced colonization (Wyszyńska et al., 2004; Buckley et al., 2010; Theoret et al., 2012; Chintoan-Uta et al., 2015). In contrast, the subcutaneous delivery route of nanoparticle encapsulated *Campylobacter* outer membrane proteins provided significant reduction in chicken colonization by *Campylobacter*, whereas the same preparation administered orally was not effective (Annamalai et al., 2013).

In contrast to many enteropathogens, *Campylobacter* produces a polysaccharide capsule (CPS). So, it was reasonable to check the efficacy of a conjugated vaccine, even though these kinds of vaccine induce an IgG, not an IgA, immune response. Conjugate vaccines are composed of capsular polysaccharide antigens covalently linked to carrier proteins which enhance the T cell-dependent response (Goldblatt, 2000). They are safe and effective, as was documented in mass routine vaccination against *Haemophilus influenzae, Neisseria meningitidis*, or *Streptococcus pneumoniae* (Trotter et al., 2008; Pace et al., 2009; Weil-Olivier et al., 2012; Davis et al., 2013). Anti-*Campylobacter* immunization with conjugated vaccines (CPS conjugated to CRM197; diphtheria toxoid) have been tested in mice, in an *Aotus nancymaae* monkey model (Guerry et al., 2012; Maue et al., 2014), and recently, also, as a chicken vaccine prototype. The induction of the bird’s immune system was dependent on the chicken immune status (SPF chicks vs. commercial broiler chicks), and a modest protection effect was observed in the case of subcutaneously immunized commercial broilers (Hodgins et al., 2015). The *Campylobacter* protein *N*-glycosylation pathway has been recently characterized in detail (Szymanski and Wren, 2005). All genes involved in the polysaccharide capsule synthesis and its transfer to the cell surface have recently been transferred to *Escherichia coli*. This cleared the way for a new technology, termed protein-glycan coupling technology (PGCT), that promises to facilitate conjugate vaccine generation that is lower cost and more repeatable than the current chemical coupling strategy (Ihssen et al., 2010; Terra et al., 2012; Cuccui et al., 2013).

So far, only limited attempts have been undertaken to explore the efficacy of non-live carriers of *Campylobacter* antigens to prevent bird colonization by *Campylobacter*. Intranasal immunization of chickens with chitosan-DNA nanoparticles, which carry the recombinant eukaryotic expression plasmid pCAGGS that encodes the major structural subunit of flagella, FlaA, resulted in modest protection against colonization (Huang et al., 2010). Also, as mentioned above, subcutaneous delivery of nanoparticle encapsulated *Campylobacter* outer membrane proteins provided significant reduction in chicken colonization by *Campylobacter* (Annamalai et al., 2013).

The aim of this study was to compare the efficacy of chicken vaccination against *Campylobacter* with non-live carriers of *Campylobacter* antigens using various means of vaccine prototype administration. We evaluated the induced immune responses and the reduction of *Campylobacter* load in bird digestive tracts after immunization with GEM particles and liposomes used as delivery vectors for two *Campylobacter* immunogenic proteins (CjaA and CjaD used together, or the chimeric rCjaAD).

## Materials and Methods

### Bacterial Strains, Plasmids, Media and Growth Conditions

Bacterial strains and plasmids used in this study are listed in **Table 1**. The *Lactobacillus salivarius* IBB3154 strain used in this study was cultured in MRS-liquid or MRS-agar (solidified with 1.5% agar) medium (Difco Laboratories, Detroit, MI, USA) at 37°C (Kobierecka et al., 2015). The *E. coli* strain TG1 was used as a host for the construction of recombinant plasmids. The *E. coli* strain Rosetta (DE3) pLysS was used to overexpress pUWM1414, pUWM1293 and pUWM1313, and *E. coli* strain BL21 (DE3) was used to overexpress pUWM1282 (Łaniewski et al., 2014). *C. jejuni* and *E. coli* strains were grown under standard conditions (Łaniewski et al., 2014) unless otherwise indicated. When needed, media were supplemented with antibiotics at the following concentrations: 30 μg ml^-1^ kanamycin and 15 μg ml^-1^ chloramphenicol, Campylobacter Selective Supplement (Oxoid) and IPTG (3 mg ml^-1^) in DMF (dimethyl-formamide).

**Table 1:** Bacterial strains and plasmids used in this study.

Strain or plasmid	Relevant phenotype(s) or genotype(s)	Source or reference(s)
**Strains**
*E. coli* TG1	*supE thi-1 Δ*(*lac-proAB*) *Δ*(*mcrB-hsdSM*)*5* (*rK^-^ mK^-^*) F′ [*traD36 proAB + lacI^q^ lacZ_M15*]	Sambrook and Russel, 2001
*E. coli* Rosetta pLysS (DE3)	F*^-^ ompT hsdS*_B_(r_B_*^-^* m_B_*^-^*) *gal dcm* (DE3) pLysSRARE (Cm^R^)	Novagen
*L. salivarius* IBB3154	Isolated from chickens	Kobierecka et al., 2015
*C. jejuni* 12/2	Wild type; isolated from a chicken; good colonizer; pUOA18 (Cm^R^)	Wyszyńska et al., 2004
**Plasmids**
pGEM-T Easy	Ap^R^; T vector for cloning PCR products	Promega
pET28a	Km^R^; *lacI*; overexpression vector	Novagen
pET22	Ap^R^; *lacI*; overexpression vector	Novagen
pBluescript II SK	Ap^R^; general cloning vector	Stratagene
pUWM1287	Source of fragment DNA encoding motif *lysM L. lactis* IL1413	This work
pUWM1293	*6xhis-lysM* fusion in pET28b	This work
pUWM1312	*6xhis-cjaA-lysM* fusion in pET28a	Kobierecka et al., 2015
pUWM1282	*6xhis-cjaD-lysM* fusion in pET28a	Kobierecka et al., 2015
pUWM1379	*6xhis-rcjaAD-6xhis* fusion in pET28a	Kobierecka et al., 2016
pUWM1414	*6xhis-rcjaAD-lysM* fusion in pET28a	This work

The 1,2-Dipalmitoyl-sn-glycero-phosphatidylcholine (DPPC), 1,2-Distearoyl-sn-glycero-phosphoethanolamine-*N*-[poly(ethyl-ene glycol)2000] (DSPE-PEG 2000) and cholesterol (Chol) were purchased from Northern Lipids, Inc. (Vancouver, BC, Canada). Sephadex G-50 fine was obtained from Sigma–Aldrich Chemie GmbH (Steinheim, Germany). All the other reagents were of analytical grade.

### General DNA Manipulations

Standard DNA manipulations were carried out as described earlier by Sambrook and Russel (2001) or according to the manufacturer’s instructions (A&A Biotechnology, Poland). DNA sequencing for cloning experiments was performed by Genomed S.A., Warsaw, Poland.

### Construction of Recombinant Plasmids for Recombinant Protein Overexpression

The LysM expression vector was constructed as follows. The LysM coding sequence was cloned from pUWM1287 to pET28b using BamHI and XhoI restriction enzymes, generating pUWM1293. Construction of the CjaA-LysM and CjaD-LysM expression vectors (pUWM1312 and pUWM1282, respectively) was described previously (Kobierecka et al., 2015). An rCjaAD-LysM expression vector was constructed as follows. The plasmid pUWM1293, was digested with XhoI and BamHI restriction enzymes and a 0.75 kb DNA fragment coding LysM was inserted into pUWM1379, generating plasmid pUWM1414 (Kobierecka et al., 2016).

Correct construction of the plasmids was verified by sequencing. Protein production was confirmed by a Western blot using previously obtained rabbit polyclonal anti-CjaD, anti-CjaA (Pawelec et al., 2000; Łaniewski et al., 2012) and rabbit polyclonal anti-LysM serum. All the recombinant plasmids encode proteins with a 6His tag fused to their N-terminus to allow purification by affinity chromatography.

### Protein Work

#### Overexpression and Purification of LysM, CjaALysM, CjaDLysM, rCjaADLysM, rCjaAD

CjaDLysM, CjaALysM, rCjaAD were overexpressed and purified as described previously (Kobierecka et al., 2015, 2016). The protein rCjaADLysM was overexpressed from an *E. coli* Rosetta (DE3) pLysS strain harboring pUWM1414, using autoinduction as described by Studier (2005). After 24 h, cultures were centrifuged and the cell pellets were suspended in 50 mM sodium phosphate, pH 8.0, 300 mM NaCl, 10 mM imidazole with 10 mg ml^-1^ lysozyme and 1 mM PMSF. Cells were disrupted by sonication. Subsequently, the cell lysates were centrifuged and the resulting supernatants were applied onto a HisTrap column (Novagen). The proteins were eluted with an imidazole gradient. LysM was overexpressed and purified from *E. coli* Rosetta (DE3) pLysS harboring pUWM1293. Expression of LysM was induced with 0.5 mM IPTG at OD_600nm_ ∼0.6 from cells growing at 18°C. After 24 h, the culture was centrifuged and the cell pellet was suspended in Cell Lysis Buffer I (50 mM Tris-HCl; pH 8.0, 1 mM EDTA, 100 mM NaCl) with 0.2 mg ml^-1^ lysozyme and 0.1 mM PMSF. After 30 min of incubation, deoxycholic acid was added. Purification and washing of inclusion bodies was done using Triton X-100. The pellet was suspended in Cell Lysis Buffer II (50 mM Tris-HCl; pH 8.0, 10 mM EDTA, 100 mM NaCl, 0.5% (v/v) Triton X-100) at a 1:9 ratio. After 5 min incubation, the suspension was centrifuged and pellet was suspended in Inclusion-body Solubilization Buffer I (50 mM Tris-HCl; pH 8.0, 1 mM EDTA, 100 mM NaCl, 8 M urea, 0.1 mM PMSF). After 1 h, Inclusion-body Solubilization Buffer II was added at a 1:9 ratio. The pH was readjusted to 10.7 using 10 N KOH. After 30 min, the pH of the solution was adjusted to eight using 12 N HCl. After 30 min suspension was centrifuged and the pellet was resuspended in Buffer B (100 mM sodium phosphate, 10 mM Tris-HCl, 8 M urea, pH 8.0) and then sonicated. Subsequently, the lysate was centrifuged and the resulting supernatant was applied onto a HisTrap column (Novagen). The proteins were eluted with decreasing pH.

Fractions containing CjaDLysM, CjaALysM, rCjaADLysM, LysM were pooled and extensively dialyzed against phosphate buffered saline (PBS). Overexpression and all purification steps were monitored by SDS-PAGE.

LysM was used for rabbit immunization. Rabbit immunization was carried out according to the ethical standards and with the approval (No. 448/2013) of the Local Ethics Committee No. 1, Warsaw, Poland.

The anti-LysM rabbit serum was specific and recognized LysM protein, as verified by Western blot analysis.

### SDS-PAGE and Western blotting

SDS-PAGE and Western blotting procedures were done by standard techniques. Blots were developed with nitro blue tetrazolium chloride/5-bromo-4-chloro-3-indolyl phosphate (Sigma–Aldrich) as a substrate, using previously obtained rabbit polyclonal anti-CjaA, anti-CjaD, anti-rCjaAD (Pawelec et al., 2000; Łaniewski et al., 2012), or anti-LysM (this work) sera as primary antibodies, and mouse anti-rabbit IgG alkaline phosphatase conjugate (Sigma–Aldrich) as secondary antibodies.

### Assessment of the Immune Responses and Chicken Protection

#### Preparation of GEM Particles

GEM particles were obtained by chemical pre-treatment *L. salivarius* IBB3154 with 10% trichloroacetic acid (TCA) as described previously (Kobierecka et al., 2015). Briefly, bacterial cells from culture with an absorbance of about 1.0 at OD_600__nm_ were collected by centrifugation and washed with phosphate-buffered saline (PBS). Next, the washed cells were resuspended in 10% TCA solution and boiled for 30 min (Bosma et al., 2006). Then, the GEM particles were washed three times with PBS. For protein binding, the prepared GEM particles were resuspended in MRS. Then 2.5 × 10^9^ of GEM particles were mixed with 2000 pmol of recombinant proteins (I. 1000 pmol CjaALysM and 1000 pmol CjaDLysM; II. 2000 pmol rCjaADLysM), incubated for 60 min, and then washed three times with PBS.

#### Preparation of Liposomes Containing rCjaAD Protein

DPPC/Chol/DSPE-PEG 2000 (5.8:4:0.2 mol/mol) liposomes were prepared using the dehydration and hydration method followed by an extrusion protocol. Briefly, 90 mg of lipids were dissolved in 4 ml of cyclohexane with 100 μl of methanol and frozen in liquid nitrogen. The sample was then freeze-dried overnight at low pressure using a Savant Modulyo apparatus (Savant, USA). Multilamellar vesicles (MLVs) were formed by hydrating the lipid film in 2.8 ml of deionized water, at 50°C. Large unilamellar vesicles (LUVs) were prepared by extrusion through Nucleopore polycarbonate filters with a pore size of 100 nm (five passes) on a Thermobarrel Extruder (Lipex Biomembranes, Vancouver, BC, Canada). The extruder was equilibrated to a temperature of 50°C prior to liposome extrusion. The mean diameter of the vesicles was determined (multimodal analysis, volume weighted) using a Zetasizer Nano-ZS (Malvern Instruments Ltd., Malvern, UK). They were generally in the size range 110–120 nm. Then, 2 ml of liposomal suspension was mixed with 1000 μl of the rCjaAD protein solution (10 mg ml^-1^). The sample was then freeze-dried overnight at low pressure using the Savant Modulyo apparatus, and the resulting lipid/protein powder was suspended in 1 ml of deionized water at 50°C and extruded through a 400 nm pore filter (five passes) as described above. The size of liposomes was usually in the range of 350–365 nm.

#### Determination of Protein Incorporation Efficiency (IE)

Non-encapsulated protein was removed from the rCjaAD-containing liposomes by size exclusion chromatography on a Sephadex G-50 mini-column (10 × 150 mm) equilibrated with 150 mM NaCl solution. The concentration of the protein was assessed by the use of modified Laurie protocol. A 30 μl liposome suspension was mixed with 70 μl of deionized water, followed by addition of 500 μl A/B solution mixture (50:1), and then 100 μl of the 2x diluted Folin-Ciocaltau reagent. Next, 60 μl of the 10% Triton X-100 solution was mixed with the sample and the sample was heated to 60°C for 30 s. and then cooled immediately in water (room temperature). The absorbance of the samples after 30 min incubation was read at 778 nm (Shimadzu UV 2401 PC spectrophotometer, Shimadzu, Japan) and the amount of protein was calculated from a previously prepared standard curve for the pure protein (5–50 μg). The incorporation efficiency (IE) was calculated as the percentage of rCjaAD protein remaining with the liposomes following elution, for the normalized lipid concentration. Lipid concentration was assessed by the modified Stewart method (Stewart, 1980).

#### Immunization and Challenge Regimens

All animal experiments were carried out according to the ethical standards and with the approval (No. 397/2012 and No. 516/2013) of the Local Ethics Committee No. 1, Warsaw, Poland. Chickens were confirmed to be culture-negative for *Campylobacter* by cloacal swabbing.

#### Per os and Subcutaneous Immunization

Hy-line chickens were obtained on the day of hatch from a local hatchery. Birds were randomly assigned to experimental groups and housed in an animal facility in separate cages for each group. Experiments were performed on chickens hatched and reared under controlled conditions from the day of hatch. The chickens were kept under controlled light (L:D 12:12) and temperature (32 ± 2°C during first week and 24 ± 2°C thereafter) conditions, with free access to the standard food and water.

Chickens deprived of food and water for 4 h were orally or subcutaneously inoculated with 2.5 × 10^9^ CFU of *L. salivarius* GEM particles with CjaALysM and CjaDLysM presenting on their surface. Booster doses were administrated 9 and 19 days after primary immunization. Following vaccination, chickens were observed for development of diarrhea and other potential adverse side effects. A group of birds inoculated with BSG was used as control. At the 30th day of life, birds were orally challenged with ∼10^4^ CFU of *C. jejuni* 12/2. At 5 and 10 days post-challenge, 5–7 birds from each group were euthanized and samples of cecum were collected. Dilutions of the contents were made in PBS and plated onto BA plates supplemented with 5% horse blood, “Campylobacter Selective Supplement (Blaser-Wang)” and chloramphenicol (15 μg ml^-1^) for enumeration of *C. jejuni*. Plates were incubated at 37°C for 48 h. Plates that were culture-negative at 48 h were reincubated for an additional 48 h. This procedure permits detection of 10^3^ CFU/g of cecal contents.

#### *In Ovo* Immunization

Animal experiments were performed using the Rosa 1 breed of chickens. This breed was created by crossing a Sussex hen with a Rhode Island Red rooster. Eighteen-day-old embryonic chicken eggs obtained from a local hatchery were inoculated with GEM particles or liposomes. Birds inoculated with PBS were used as a control group. Using a needle, 0.1 ml of inoculum was injected into the amniotic fluid. Hatched chickens of each group were placed in separate cages and provided with *ad libitum* food and water during the experimental period.

At 2 weeks of age, birds were orally challenged with ∼10^6^ CFU of *C. jejuni* strain 12/2. At weeks 1 and 2 post-challenge, 6 birds (from each group) were euthanized and samples of cecum were collected. Dilutions of the contents were made in PBS and plated onto BA plates supplemented with 5% horse blood, “*Campylobacter* Selective Supplement (Blaser-Wang)” and chloramphenicol (15 μg ml^-1^) for enumeration of *C. jejuni*. Plates were incubated at 37°C for 48 h. Plates that were culture-negative at 48 h were reincubated for an additional 48 h. This procedure permits detection of 10^3^ CFU/g of cecal contents.

Additionally, to monitor the humoral immune response, six birds from each group were sacrificed on days 7, 14, 21, and 28 post-hatch and samples of gut secretion were collected for the post-mortem examination. Secretory IgA antibodies were extracted from lower parts of the intestine with PBS containing 0.05% Tween 20 and soybean trypsin inhibitor (0.1 mg ml^-1^) (dilution 1:10). Samples were shaken for 2 h at 4°C, centrifuged at 20,000 × *g* for 30 min at 4°C, and afterward the supernatant was collected and stored at -20°C.

### Enzyme-Linked Immunosorbent Assay (ELISA)

The 6xHis-tagged rCjaAD protein purified as described above was also used as a coating antigen. The levels of antibody against rCjaAD protein in chicken intestinal secretions were quantified by ELISA. Briefly, 96-well Maxisorp plates (Nunc, Rochester, NY, USA) were coated with purified rCjaAD protein (5 μg per well) in PBS and incubated overnight at 4°C. Then, plates were blocked for 1 h at 37°C with PBS containing 0.1% Tween 20 (Sigma–Aldrich) and 1% bovine serum albumin (BSA), washed three times with PBS containing 0.1% Tween 20 (Sigma–Aldrich) and incubated for 1 h at room temperature with the intestinal secretion samples (1:10). Goat anti-chicken IgA horseradish peroxidase conjugate (Thermo Fisher, Scientific) was employed to detect chicken IgA that bound to *Campylobacter* antigens. The plates were developed with 3,3′,5,5′-tetramethylbenzidine (Sigma–Aldrich), according to the manufacturer’s directions. The reaction was stopped with 3M H_2_SO_4_ and optical density was determined at *A* 490 using an ELISA reader (Tekan). Each sample was analyzed in triplicate.

### Statistical Analysis

Statistical analyses of the colonization results and ELISA test were performed using STATISTICA 10PL software (StatSoft, USA). The significance of differences between the obtained values was appraised using the Kruskal–Wallis test. Any *p*-values <0.05 were considered significant.

## Results

### Oral and Subcutaneous Immunization with GEM Particles Presenting CjaA and CjaD Proteins – Protection Analysis

Two highly immunogenic, extracytoplasmic and conserved *C. jejuni* proteins — CjaA (Cj0982c in the genome of *C. jejuni* NCTC11168) and CjaD (Cj0113 in the genome of *C. jejuni* NCTC11168), which are often tested as candidates for chicken anti-*Campylobacter* vaccination – were chosen for this study. The immunogenicity of CjaA and CjaD has been documented by our research group, as well as by others (Wyszyńska et al., 2004; Buckley et al., 2010; Layton et al., 2011; Clark et al., 2012; Hoppe et al., 2012). Recently we have shown that GEM particles (Gram-positive Enhancer Matrix), from TCA-pretreated *L. salivarius*, can act as a surface display platform for *C. jejuni* antigens (Bosma et al., 2006; Kobierecka et al., 2015). In this study we tested the efficacy of chicken immunization with GEMs presenting CjaA and CjaD, using two routes of vaccine administration. Both proteins, fused with the PA binding domain of the *L. lactis* peptidoglycan hydrolase, AcmA, that contains three lysine motifs (LysM), were obtained using an *E. coli* expression system and were purified by affinity chromatography. Both chimeras reacted with specific rabbit anti-LysM serum (**Supplementary Figure S1**). Chimeras were bound to GEM particles as described previously (Kobierecka et al., 2015). The binding efficiency was determined by Western blot analysis using specific rabbit anti-CjaA and anti-CjaD sera (data not shown). First, we examined whether oral or subcutaneous immunization resulted in reduction of the *Campylobacter* load in bird intestinal tracts. For oral immunization, 1-day old chickens were immunized with GEM particles presenting CjaA and CjaD. A groups of birds inoculated with BSG or GEM particles were used as control (details are given in the Section “Materials and Methods”).

**FIGURE 1:**
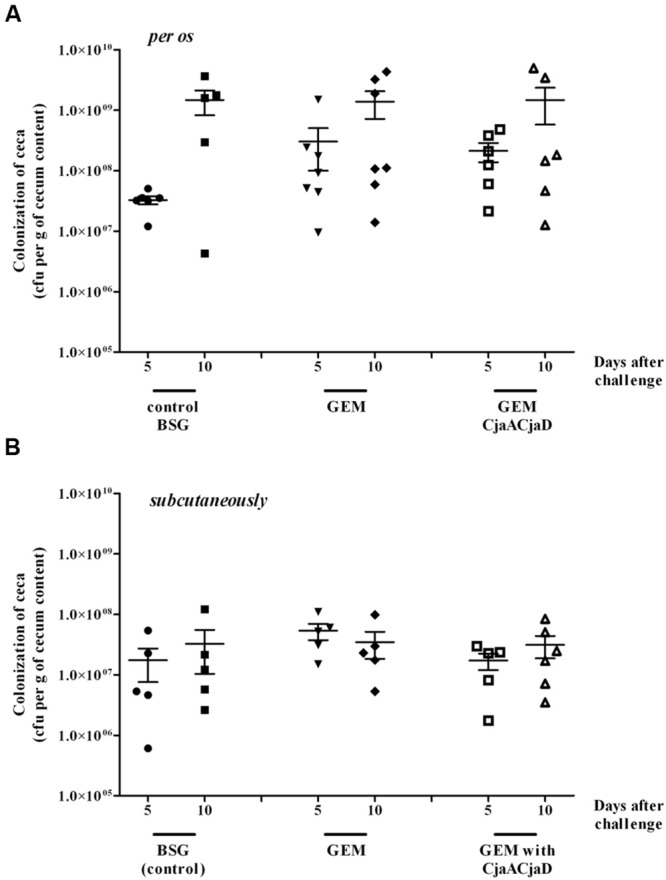
**Colonization of chickens vaccinated with GEM particles or GEM particles presenting CjaA-LysM and CjaD-LysM on their surface and then given a *Campylobacter jejuni* challenge.** Chickens were orally **(A)** or subcutaneously **(B)** given three doses of the vaccine at 1, 9, and 19 days after hatch and challenged with *C. jejuni* 12/pUOA18 at the 30th day of life. Control birds were given BSG (PBS with 0,01% gelatin). Viable *C. jejuni* cells were recovered from the ceca of chickens at specified days after challenge. Bacterial recoveries represent colonization levels of 6 or 7 birds per time interval. The geometric mean for each group is denoted by bars. No significant differences (*p* < 0.05) between groups were seen.

Chickens were boosted with the same doses of identical GEM particles at 9 and 19 days post-hatch and were orally challenged with ∼10^4^ CFU of *C. jejuni* wild-type strain 12/2 at the 30th day of life. The level of colonization was evaluated at 5 and 10 days post challenge (**Figure 1A**). For subcutaneous immunization, the same scheme was applied (**Figure 1B**). We found that neither the oral nor the subcutaneous route of vaccination resulted in a protective effect against bird intestinal tract colonization by wild type *C. jejuni* strain.

### *In Ovo* Immunization Using GEM Particles and Liposomes as Vectors for *Campylobacter* Antigens

*In ovo* chicken vaccination has been a common practice in the poultry industry for many years to protect birds, mainly against viral diseases such as Marek’s disease, infectious bursal disease or Newcastle disease (Negash et al., 2004). Given that chickens develop certain immunologic functions before hatching, we decided to evaluate the efficacy of *in ovo* chicken immunization against *C. jejuni* with two non-live carriers of *Campylobacter* antigens: GEM particles and neutral liposomes. To the best of our knowledge, liposomes, which have been studied as a delivery system for many vaccine formulations, have so far not been tested for *in ovo* chicken immunization. Instead of employing the two separate antigens, CjaA and CjaD, we used the recently generated hybrid protein rCjaAD for the immunizations with either GEM particles or liposomes as carriers. rCjaAD is a CjaA that presents three selected CjaD epitopes on its surface. It has been shown the specific antibodies obtained by rabbit immunization with rCjaAD recognize both of the native CjaA and CjaD proteins produced by wild type *Campylobacter* (Kobierecka et al., 2016).

### Characterization of the GEMs-rCjaAD Complexes

To ensure its binding to GEMs, rCjaAD was fused to the PA binding domain of the *L. lactis* peptidoglican hydrolase, AcmA, which contains LysM motifs (Bosma et al., 2006). The rCjaAD-LysM chimera, additionally equipped with a 6-His tag, was obtained using an *E. coli* expression system and affinity chromatography purification. Its proper conformation was confirmed by Western blot analysis using specific rabbit anti-CjaA, anti-CjaD and anti-LysM antibodies (**Supplementary Figure S2**). rCjaAD was bound to the surface of GEMs particles as described previously (Kobierecka et al., 2015). The binding efficiency was determined by Western blot analysis using specific rabbit anti-CjaA and anti-CjaD sera (data not shown) and by immunofluorescence (**Supplementary Figure S3**).

### Characterization of Liposome-rCjaAD Complexes

The rCjaAD protein employed in this experiment was obtained as described above using an *E. coli* expression system and affinity chromatography purification. After addition of the protein solution to the unilamellar liposome suspension, the mixture immediately became less transparent, with no essential liposomes size increase, suggesting a strong protein bilayer interaction and protein incorporation into the bilayer. Measurement of protein incorporation efficiencies supports this presumption because, despite the protein incorporation method (data not shown), this parameter varied only from 96 to 98%, indicating that the protein is incorporated into the bilayer, not encapsulated within the liposome aqueous interior. Such protein localization in the neutral liposomes further facilitates liposome/protein–cell interactions. Attempts to use cationic liposomes instead of neutral ones (data not shown), which contained 20% cationic lipid (DOTAP), resulted in strong electrostatic complex formation between large unilamellar liposomes (110 nm) and rCjaAD protein, leading to liposome aggregation and fusion. Attempts to decrease the size of the aggregated liposomes was not successful, either by extrusion or mild sonication (liposomes stacked on the polycarbonate filter during extrusion).

### Chicken Immunization *In Ovo*

Two groups of 18-day-old embryonated chicken eggs were immunized with GEM particles or liposomes carrying *Campylobacter* antigen. A group of birds inoculated with PBS was used as a control. The details of the immunization procedure are given in materials and methods section. The protective effect of *in ovo* vaccination was assessed by a plating method after oral challenge with 1 × 10^6^ bacterial cells of a broiler-isolated *C. jejuni* strain. The *C. jejuni* strain used for the challenge experiment was labeled with the pUOA18 plasmid containing a *cat* gene.

**FIGURE 2:**
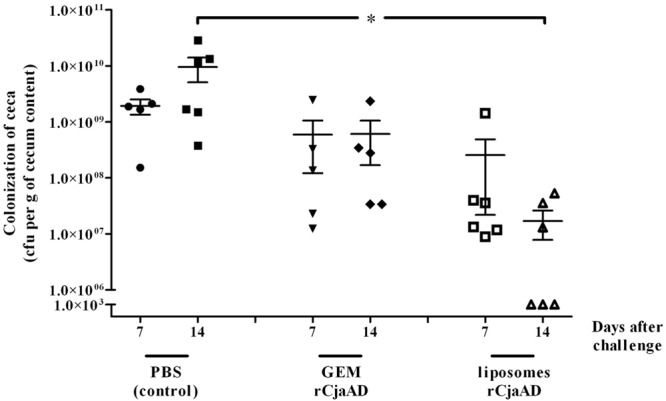
**Colonization of chickens vaccinated *in ovo* with: GEM particles presenting rCjaAD-LysM on their surface or liposomes containing rCjaAD, and then given a *C. jejuni* challenge.** 18-day-old embryonated chicken eggs were inoculated with 0.1 ml GEM particles or liposomes and challenged with *C. jejuni* /pUOA18 on day 14 of life. Control birds were given PBS. Viable *C. jejuni* cells were recovered from the ceca of chickens 7 and 14 days after challenge. Bacterial recoveries represent colonization levels of 5 or 6 birds per time interval. The geometric mean for each group is denoted by bars. Asterisks indicate significant differences (*p* < 0.05) between analyzed groups and control group.

We found that both GEMs particles and liposomes with rCjaAD reduced the level of birds’ caeca colonization by wild type *Campylobacter* as compared to the control group (**Figure 2**). The mean CFU/gram of cecal content observed in the group that received GEMs presenting rCjaAD was about 1 × 10^9^, whereas the mean level of colonization in the control group was ∼1 × 10^10^ CFU/gram. The protective effect was even more significant for the group immunized with rCjaAD delivered by liposomes. The mean CFU/gram of cecal content observed after 2 weeks in the group that received liposomes containing rCjaAD was about 2 × 10^7^. And, importantly, three out of six chickens were colonized below detection level (10^3^ CFU/g of cecal contents).

**FIGURE 3:**
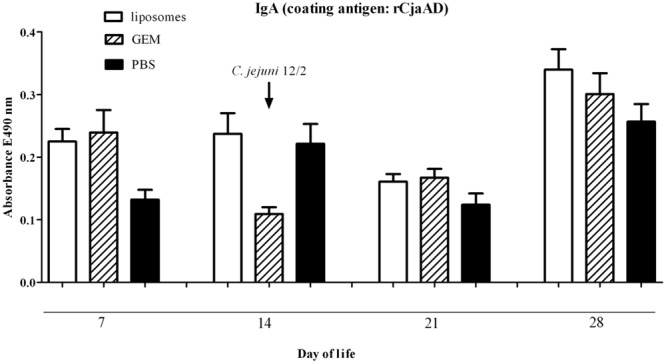
**Immune responses of chickens vaccinated *in ovo* with GEM particles presenting rCjaAD-LysM on their surface or liposomes containing rCjaAD.** Levels of mucosal sIgA antibodies specifically recognizing rCjaAD antigen were determined by ELISA. Eighteen-day-old embryonated chicken eggs were inoculated with 0.1 ml GEM particles or liposomes and challenged with *C. jejuni* /pUOA18 on day 14 of life. Control birds were given PBS (solid bars). Intestinal samples were collected at the specified days of chicken life. Purified rCjaAD protein was used as a coating antigen. Intestinal secretion samples were diluted 1:10. Absorbance values represent a mean of 5 or 6 birds ±SD per time interval. No significant differences (*p* < 0.05) between analyzed groups and control groups were seen.

The level of the specific intestinal IgA against rCjaAD was measured for chickens at days 7, 14, 21, and 28 post-hatch. The data show that *in ovo* immunization stimulated the gut-associated immune system, and the effect was more marked for vaccination using liposomes as the delivery vector (**Figure 3**). The high response observed in the control group (14 days after hatch) resulted from a high IgA level detected in the intestinal sample of one bird.

The median reduction of *C. jejuni* cecal contents was 1 log_10_ for *in ovo* immunization with GEM particles containing rCjaAD and ∼2 log_10_ for *in ovo* immunization with liposomes containing rCjaAD. The efficacy of *in ovo* immunization with non-live carriers may be increased by booster immunization after hatching with vectors known to induce a mucosal immune response, such as *Lactobacillus* spp. or attenuated *Salmonella* strains carrying *Campylobacter* antigens. Our results provide evidence that *in ovo* chicken vaccination using appropriate carriers and recombinant antigens may be an efficient way to reduce the level of chicken colonization by *Campylobacter* and thus reduce the burden of human campylobacteriosis.

## Discussion

Campylobacteriosis, caused mainly by *C. jejuni*, is still one of the most common food-borne human illnesses worldwide, and contaminated poultry meat is considered to be the main risk factor of human infections. Various vaccine strategies, including parenteral, oral and nasal routes using different antigens, have recently been evaluated in experimental studies to decrease the level of chicken colonization by the *C. jejuni* pathogen in order to ease this serious health problem. However, knowledge concerning the interaction between *C. jejuni* and its hosts is still limited, and we still do not understand what kind of immune response is needed to combat this pathogen. It is still debatable whether we can consider *Campylobacter* as a chicken commensal organism since infection of birds does not provoke signs of pathology, but does induce an inflammatory response. Additionally, *Campylobacter* infection has an impact on bird’s intestine functioning by influencing on specific gene expression and by inducing histomorphological changes of epithelium and modulating its barrier function (Awad et al., 2014, 2015a,b). Moreover, the observation that the immune response to infection is dependent on the breed of broiler chicken has led to substantial confusion (Humphrey et al., 2014).

To better understand the chicken immune response and develop an efficient strategy to combat chicken colonization by *Campylobacter*, this study has evaluated the protective effect of chicken vaccination using non-live vectors (GEM particles and liposomes) harboring *Campylobacter* antigens delivered by various routes. Two immunogenic *Campylobacter* antigens (CjaA and CjaD, previously used by us and others in experimental chicken oral vaccination studies), were employed (Wyszyńska et al., 2004; Buckley et al., 2010; Layton et al., 2011). Assuming that GEM particles should be taken up by epithelium M cells and based on the results of rabbit oral mucosal immunization with GEMs presenting *Plasmodium falciparum* surface antigen MSA2, we tested the efficiency of oral chicken immunization with GEMs presenting *C. jejuni* antigens (Ramasamy et al., 2006). As several attempts have been also undertaken to immunize chickens against *Campylobacter* with different protein antigens by a parenteral route of administration, we also used GEMs decorated with CjaA and CjaD for subcutaneous vaccination. Chicken immunization with GST-Dps or GST-CjaA proteins combined with adjuvant does not provide protection, whereas immunization with hybrid protein consisting of selected parts of surface–exposed colonization proteins (CadF-FlaA-FlpA; named SECPs) combined with adjuvant resulted in significant protection (Theoret et al., 2012; Neal-McKinney et al., 2014). However, it should be noted that the chickens used in these two experiments differed considerably in their immunological status (1-day-old chicks vs. specific-pathogen-free chicks). GEM particles are mainly composed of cell wall peptidoglycan. Given that peptidoglycan is a known ligand of TLR2 receptors, it should act as an immunostimulator of the innate immune system (Keestra et al., 2013). Thus, the subcutaneous immunization using GEMs that present CjaA and CjaD was not combined with extra adjuvant. Neither oral nor subcutaneous vaccination with GEMs presenting CjaA and CjaD resulted in chicken protection against *Campylobacter*, even though the same antigens administered by the oral route, using attenuated *Salmonella* as a delivery vector, produced modest or even high levels of protection (Wyszyńska et al., 2004; Buckley et al., 2010; Layton et al., 2011). It is likely that, in the case of oral immunization, both antigens displayed on the surface of GEM particles were degraded in the bird gut, so only small amount of antigens were processed by the intestinal APC cells. The chicken lines used in experiments need to be scrutinized because significant genetic differences among them, as well as differences in their gastrointestinal microbiota conditioned by diet, may significantly influence the immune responses (Rubin et al., 2010; Yeoman et al., 2012; Pan and Yu, 2014; Schokker et al., 2015). Data from oral chicken vaccination against *Campylobacter* has indicated that the intestinal mucosal immune response to produce specific sIgA, played a crucial, though not sufficient, role in a bird’s protection (Hermans et al., 2014). So far, the chicken cellular response to *Campylobacter* infection remains undefined (Wigley, 2013). Though *Campylobacter* had been thought a human pathogen that was incapable of entering intestinal epithelium cells, recent evidence shows that *Campylobacter* can transmigrate across the gut epithelial barrier, invade epithelial cells from the basolateral side and survive in specific *Campylobacter*-containing vacuoles (CCV) (Watson and Galan, 2008; Backert et al., 2013; Bouwman et al., 2013). *Campylobacter’s* interaction with the chicken immune system and with chicken intestinal epithelium cells remains poorly understood (Wigley, 2013). *Campylobacter* can invade primary chicken epithelial cells or chicken hepatocellular epithelial cells, though with low efficiency (LMH) (Byrne et al., 2007; Larson et al., 2008). So the issue whether cellular immune responses may play a role in chick protection against *Campylobacter* remains elusive.

Chicken *in ovo* immunization has long been widely used by the poultry industry to prevent viral diseases (Negash et al., 2004). The *in ovo* route of vaccine administration is easy to use for mass vaccination, and it is more precise and more efficient than post-hatch immunization by spray or drinking water. Many new vaccine formulations are under extensive investigation in order to assess their efficacy in *in ovo* immunization They include immunization with plasmid DNA, live attenuated viruses or using viral vectors as antigen carriers (Toro et al., 2007; Bande et al., 2015). To the best of our knowledge, the present work is the first to analyze the effect of chicken immunization *in ovo* with a subunit vaccine prototype against bacterial pathogens. The only experiment to estimate the results of *in ovo* vaccination against *Campylobacter* infection was performed 40 years ago with killed, whole bacterial cells. The data presented at that time indicated that *in ovo* vaccination induced a mucosal immune response (Noor et al., 1995). For our *in ovo* administration, rather than using two separate antigens, we used the hybrid rCjaAD protein, which is an engineered CjaA presenting CjaD epitopes. As previously shown, antibodies against rCjaAD recognize both native CjaA, as well as native CjaD, protein, which confirms its usefulness for vaccination (Kobierecka et al., 2015). Experiments are in progress to generate hybrid CjaA proteins presenting more epitopes originating from other conserved immunogenic proteins (Hoppe et al., 2013; Hermans et al., 2014). For evaluation of the effect of *in ovo* immunization, we used GEM particles and neutral liposomes as carriers for *Campylobacter* antigens. To the best of our knowledge, liposomes, which have been studied as adjuvants or delivery system in many vaccine preparations, so far have not been tested for *in ovo* chicken immunization (Watson et al., 2012; Schwendener, 2014). The antigens can be chemically linked to the liposome surface, bound with the lipid bilayer by electrostatic or hydrophobic interactions, or encapsulated in the liposome water compartment (Taneichi et al., 2010). In our work, we observed a strong interaction between liposomes and the rCjaAD protein, resulting in nearly 100% protein accommodation in bilayer structure. The observed anti-rCjaAD activity indicates that the protein epitopes responsible for an immunological response are at least partially exposed to immunocompetent cells and confirms that the protein possesses hydrophobic domains that are able to incorporate in the lipid bilayer. In general, protein adsorption by electrostatic interaction or binding produces higher immunization than protein encapsulation within the liposomal interior. Since promising results have been achieved with liposomal vaccination by the rCjaAD protein, several different approaches can be proposed. For example, comparison of cationic, anionic and neutral liposomes will explore the importance of charge on the immunization process. Also, incorporation of extra adjuvants like monophosporyl lipid A or alluminia salts may lead to a more complex immunological response that offers a longer lasting effect. Both vaccine prototypes were introduced into the embryo amniotic sac at day 18 of embryonation, when the chicken embryo immune system is capable of responding to administered antigen. The process should induce the gut-associated lymphoid tissues (Negash et al., 2004). Preliminary data on the level of specific anti-rCjaAD intestinal IgA confirm some gut-associated lymphoid induction. In the *in ovo* vaccination experiments, commercial broiler Rosa chicks were employed. The level of their intestinal track colonization was higher than those previously described by us and others. Why the commercial broiler Rosa chickens are so susceptible to *Campylobacter* colonization remains unclear. However, regardless of the employed carrier, *in ovo* immunization resulted in significant reduction of colonization. The median reduction in *C. jejuni* cecal contents was about 1.0 log_10_ for *in ovo* immunization with GEM particles containing rCjaAD and about 2 log_10_ for *in ovo* immunization with liposomes containing rCjaAD. The observed differences between the reduction of colonization achieved by vaccination with the two vectors (GEMs vs. liposomes) may suggest that liposomes are more efficiently taken in by APC cells. Additionally, as demonstrated, rCjaAD antigen intercalates into the lipid bilayer of the liposomes, and this may protect it from degradation. More experiments are needed to evaluate the reduction of the level of chicken colonization by *Campylobacter* by a liposome-based vaccine formulation when lower *Campylobacter* doses are used for challenge.

Overall, the presented data shows that it is worthwhile to explore the efficiency of using a subunit vaccine composed of *Campylobacter* recombinant hybrid protein delivered by non-live vectors and an *in ovo* route of vaccination to combat bird colonization by *Campylobacter*. However, it should be emphasized that routes of immunization using GEM particles as a vehicles cannot be directly compared as various chickens’ lines were used in these two sets of experiments. Nevertheless, *in ovo* vaccination posses some advantages over other routes of immunization. Firstly, it is more relevant for practical use by the poultry industry. Secondly, there are several strategies that allow incorporation of extra adjuvant molecules into the liposome, which may help modulate the strength and the type of immune response. Moreover, *in ovo* vaccination may be additionally combined with post-hatch boosting using live *Lactobacillus* isolated from the chicken gastrointestinal tract or attenuated *Salmonella* strains as delivery vectors (Wyszyńska et al., 2004; Łaniewski et al., 2012). The effect of *in ovo* immunization may be also intensified by blocking *Campylobacter* adhesion to avian mucin or by increasing activity of chicken’s defensins (Struwe et al., 2015).

## Author Contributions

EJ-K, AKW and PAK conceived and designed the study. PAK, AKW, JG, AW, OW, and KD carried out the laboratory work. AKW, PAK, MK, RG, IA carried out animal experiment. EJ-K, PAK, and AKW analyzed the data. EJ-K, AKW, JG and PAK wrote the manuscript. All authors read and approved the final manuscript.

## Conflict of Interest Statement

The authors declare that the research was conducted in the absence of any commercial or financial relationships that could be construed as a potential conflict of interest.

## Supplementary Material

FIGURE S1 **Western blot analysis of the specificity of anti-LysM serum.** Protein extracts purified by affinity chromatography were separated by 12% SDS-PAGE under reducing conditions and probed with anti-LysM antibodies. Lanes: 1 – 6xHis-LysM, 2 – 6xHis-CjaA-LysM; 3 – 6xHis-CjaD-LysM; M – protein molecular-weight marker.

FIGURE S2 **Western blot analysis of the specificity of 6xHis-rCjaAD-LysM.** Proteins were separated by 12% SDS-PAGE under reducing conditions and probed with: **(A)** anti-LysM, **(B)** anti-CjaA, and **(C)** anti-CjaD antibodies. Lanes: 1 – purified protein 6xHis-rCjaAD-LysM, M – protein molecular-weight marker.

FIGURE S3 **Localization of rCjaADLysM fusion protein on TCA-pretreated *L. salivarius*.** Fusion protein were visualized with anti-rCjaAD antibodies that were further detected with goat anti-rabbit IgG Alexa Fluor A488. Fluorescence was visualized with a NIKON A1R MP microscope. The bar represents 2 μm.
