# Supplementary figures and images for: Chicken Anti-Campylobacter Vaccine – Comparison of Various Carriers and Routes of Immunization
Source: Front Microbiol. 2016 May 19;7:740. doi: 10.3389/fmicb.2016.00740 (PMC4872485; doi:10.3389/fmicb.2016.00740)

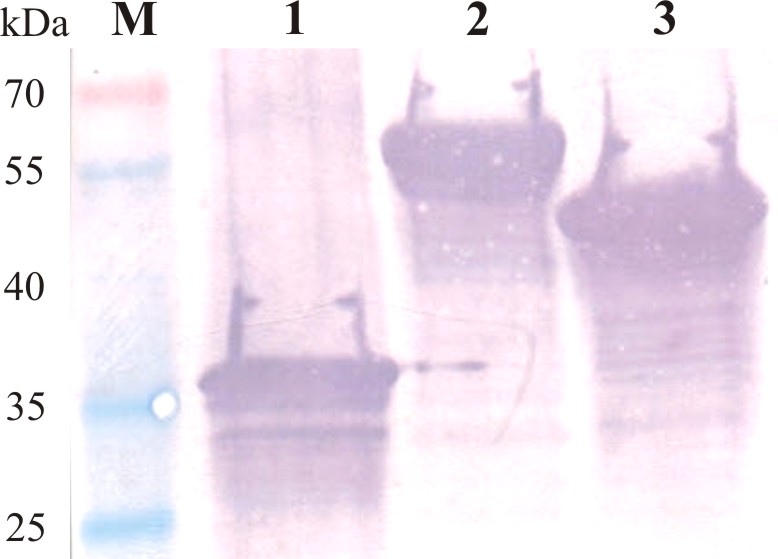

Supplement: FIGURE S1 — Western blot analysis of the specificity of anti-LysM serum. Protein extracts purified by affinity chromatography were separated by 12% SDS-PAGE under reducing conditions and probed with anti-LysM antibodies. Lanes: 1 – 6xHis-LysM, 2 – 6xHis-CjaA-LysM; 3 – 6xHis-CjaD-LysM; M – protein molecular-weight marker. [file Image_1.JPEG]

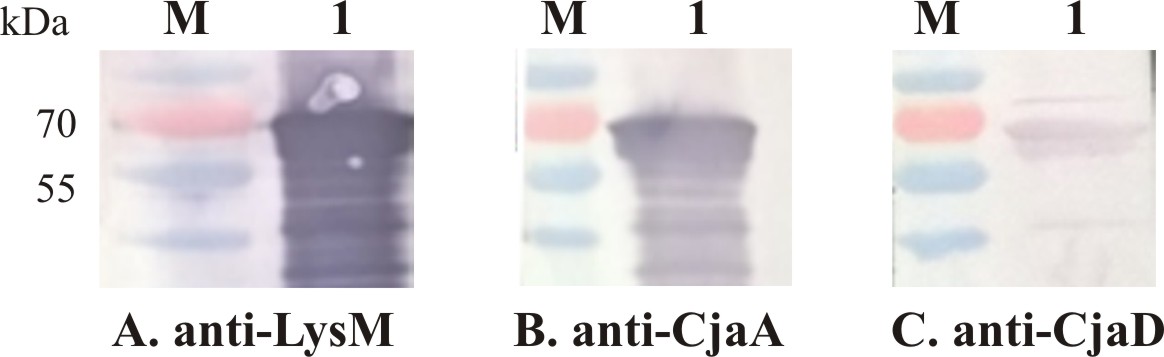

Supplement: FIGURE S2 — Western blot analysis of the specificity of 6xHis-rCjaAD-LysM. Proteins were separated by 12% SDS-PAGE under reducing conditions and probed with: (A) anti-LysM, (B) anti-CjaA, and (C) anti-CjaD antibodies. Lanes: 1 – purified protein 6xHis-rCjaAD-LysM, M – protein molecular-weight marker. [file Image_2.JPEG]

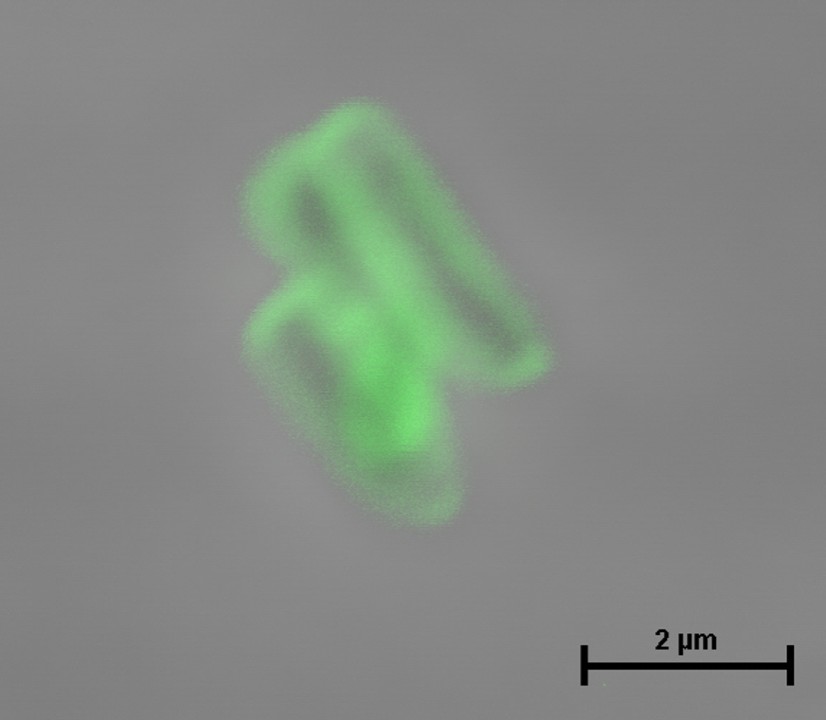

Supplement: FIGURE S3 — Localization of rCjaADLysM fusion protein on TCA-pretreated L. salivarius. Fusion protein were visualized with anti-rCjaAD antibodies that were further detected with goat anti-rabbit IgG Alexa Fluor A488. Fluorescence was visualized with a NIKON A1R MP microscope. The bar represents 2 μm. [file Image_3.JPEG]
